# Factors Influencing Clinical and Radiological Response in Perianal Disease: Results from a Real-World Cohort Treated with Anti-TNF Therapy

**DOI:** 10.3390/jcm15052001

**Published:** 2026-03-05

**Authors:** Clara Amiama Roig, Cristina Suárez-Ferrer, José Luis Rueda García, Laura García Ramírez, María Sánchez Azofra, Eduardo Martín-Arranz, Joaquín Poza Cordón, Jesús Noci, Carmen Amor Costa, Irene González Díaz, María Dolores Martín-Arranz

**Affiliations:** 1 Inflammatory Bowel Disease Unit, Gastroenterology Department, Hospital Universitario La Paz, 28046 Madrid, Spaineiiclapaz@gmail.com (L.G.R.); mmartinarranz@salud.madrid.org (M.D.M.-A.); 2 IdiPAZ Study Group for Immune-mediated Gastrointestinal Diseases, La Paz Institute for Health Research (IdiPAZ), 28046 Madrid, Spain; 3 Gastroenterology Department, Hospital Universitario La Paz, 28046 Madrid, Spain; 4 School of Medicine, Univesidad Autónoma de Madrid, 28049 Madrid, Spain

**Keywords:** perianal Crohn’s disease, anti-TNF therapy, real-world cohort, clinical response, radiological response, therapeutic drug monitoring, treatment predictors, long-term outcomes

## Abstract

**Background**: Perianal Crohn’s disease (PD) remains a major therapeutic challenge, with heterogeneous responses to anti-TNF therapy and limited real-world data on predictors of long-term outcomes. This study aimed to evaluate clinical and radiological response to anti-TNF therapy initiated exclusively for PD and to identify factors associated with treatment response. **Methods:** A retrospective study was conducted in a cohort of 65 patients with PD treated with anti-TNF. The primary endpoint was clinical response assessed at weeks 24, 52, and 60 months. It was defined as a ≥50% reduction in drainage, and remission as complete absence of drainage. Radiological response was assessed by magnetic resonance imaging at the same time points whenever feasible. Multivariate logistic regression analyses were performed to identify independent predictors of response. **Results:** At week 24, 84.6% of patients achieved a clinical response, while radiological response was observed in 30.8%. At week 52, clinical and radiological response rates were 80.0% and 52.3%, respectively. At 60 months, 61.5% maintained clinical response and 46.1% radiological response. Among patients who responded at week 24, 90.7% maintained response at week 52, with a secondary loss of response rate of 9.3%. Multivariate analysis identified absence of antineutrophil cytoplasmic antibodies (ANCA) as an independent predictor of clinical response at week 52 (OR 0.06, 95% CI 0.006–0.59; *p* = 0.01). No significant associations were observed between anti-TNF serum levels and clinical or radiological outcomes. **Conclusions**: In this real-world cohort of patients initiating anti-TNF exclusively for PD, early response (week 24) emerged as a potential marker of long-term outcomes, highlighting the importance of early reassessment and individualized therapeutic strategies.

## 1. Introduction

Perianal disease (PD) associated with Crohn’s disease (CD) represents one of the most complex and debilitating manifestations of inflammatory bowel disease (IBD), with a significant clinical, functional, and psychological impact [1,2]. The increased risk of surgery and the frequent need for therapeutic escalation [3,4] make the management of PD a therapeutic challenge.

Optimal management requires a multidisciplinary approach and the sequential or concomitant use of surgery, antibiotics, immunosuppressants, and biologic therapies [5,6,7,8]. The introduction of anti-tumor necrosis factor (anti-TNF) agents represented a paradigm shift in treatment, demonstrating efficacy in fistula closure and improvement in both controlled trials and real-world studies [9,10,11,12,13]. Nevertheless, treatment response is heterogeneous: a substantial proportion of patients fail to achieve an adequate response, many require treatment intensification, and there are significant rates of both primary non-response and secondary loss of response. Identifying baseline and dynamic factors that predict an unfavorable course is, therefore, a critical clinical need.

Several studies have suggested that serum anti-TNF concentrations correlate with improved outcomes, particularly during induction therapy, showing that higher drug levels are associated with a greater likelihood of fistula closure [10,11,14]. However, the applicability of these findings to routine clinical practice remains limited.

Evidence from real-world cohorts with long-term follow-up and systematic pharmacokinetic monitoring is scarce. Most available studies focus on early outcomes (weeks 12–14) and lack structured long-term radiological assessment. In addition, patients in whom anti-TNF therapy is initiated for PD are often analyzed together with those treated for luminal disease, thereby limiting the interpretability of the results.

Therefore, the present study analyzed a real-world cohort of patients with PD treated with anti-TNF therapy, including exclusively those in whom anti-TNF treatment was initiated specifically for a perianal indication and strictly excluding cases in which the primary indication was luminal disease or extraintestinal manifestations. The aim was to more precisely characterize therapeutic response and to explore different factors, beyond drug levels, that may influence clinical and radiological outcomes both during induction and in the long term.

## 2. Materials and Methods

A retrospective observational study was conducted, including all patients with CD and PD followed at the IBD Unit of La Paz University Hospital between March 2012 and February 2023, in whom anti-TNF therapy was initiated exclusively for a perianal indication.

All cases in which anti-TNF therapy had been initiated for luminal disease activity or extraintestinal manifestations were excluded, as well as patients without pharmacokinetic measurements during induction, those who lost to follow-up, with poor treatment adherence, or with incomplete medical records.

For each patient, demographic variables, disease phenotype according to the Montreal classification, smoking status, serological markers (ASCA and ANCA), and PD characteristics were recorded. These included fistula type according to the Parks classification, number of fistulous tracts, presence of abscesses, surgical interventions, and examinations under anesthesia (EUA), seton placement, and previous or concomitant therapies. Trough serum levels of infliximab (IFX) or adalimumab (ADA) measured during induction (weeks 2 and 6) and during maintenance (weeks 24, 52, and 60 months) were also collected, in accordance with routine clinical practice at our center.

The primary endpoint of the study was clinical response to anti-TNF therapy, assessed at weeks 24, 52, and 60 months after treatment initiation. Clinical assessment was based on the evaluation of fistula drainage during standardized outpatient visits. Clinical response was defined as a ≥50% reduction in fistula drainage compared with the baseline. Clinical remission was defined as the complete absence of drainage on physical examination and/or closure of the external fistula opening (EFO).

The secondary endpoint was radiological response, assessed by pelvic magnetic resonance imaging (MRI). All MRI studies were interpreted by radiologists specialized in PD. Radiological response was defined as the absence of T2 hyperintensity and gadolinium enhancement on T1-weighted images, in the absence of abscess and rectal inflammation.

Clinical evaluations were systematically performed at weeks 24, 52, and 60 months. MRI assessments were performed according to clinical availability, prioritizing the same time windows whenever possible, but were not protocol-mandated at each visit. Consequently, MRI data were available for 35/65 patients at week 24 and for 40/65 patients at both week 52 and 60 months.

Surgical management followed the unit’s established protocols, including abscess drainage, sequential placement or removal of setons, and EUA performed in one or two stages, at the discretion of the responsible surgeon. Anti-TNF therapy was initiated at label-recommended dosing regimens. In patients with insufficient perianal response, dose intensification was permitted at the discretion of the treating physician. For IFX, escalation to 10 mg/kg and/or administration every 6 or 4 weeks was allowed. For ADA permitted regimens include 40 mg weekly, 80 mg every 14 days, or 80 mg weekly.

Drug levels were determined immediately prior to infusion or injection (trough levels) using an enzyme-linked immunosorbent assay (ELISA) with Promonitor^®^ ELISA kits (Grifols, Barcelona, Spain). Drug concentrations were expressed in µg/mL. Importantly, as long as detectable serum levels of anti-TNF are present, anti-drug antibodies will not be identified by the ELISA method employed, since they remain bound to the drug.

Statistical analysis was performed using Stata software, version 16. A descriptive analysis of demographic and IBD-related characteristics was conducted. Continuous variables with a normal distribution were expressed as means and standard deviations, while non-normally distributed continuous variables were reported as medians with interquartile ranges. Categorical variables were expressed as percentages. Normally distributed quantitative variables (assessed using the Shapiro–Wilk test) were compared using Student’s *t* test. Non-parametric tests were used when normality was not met: the Mann–Whitney U test for independent groups and the Wilcoxon test for paired data. Categorical variables were analyzed using the chi-square test. Multivariate logistic regression models were constructed to identify independent predictors of clinical and radiological response, adjusting for potential confounders, and to estimate odds ratios (ORs) with 95% confidence intervals (CIs). Covariates with *p* < 0.1, plus clinically relevant covariates according to previous literature, were included in the multivariate logistic regression. The discriminative ability of drug levels to predict response was assessed using receiver operating characteristic (ROC) curves, calculating the area under the curve (AUC) and the optimal cutoff value according to the Youden index. Statistical significance was set at *p* < 0.05.

Artificial intelligence assistance: During manuscript preparation, ChatGPT (OpenAI, version GPT-5.2) was used exclusively to assist in generating a graphical representation of the study data (Figure 2). The authors reviewed and validated all AI-generated content.

The study was approved by the Clinical Research Ethics Committee of La Paz University Hospital (PI-5531).

## 3. Results

### 3.1. Study Population

**Figure 1:**
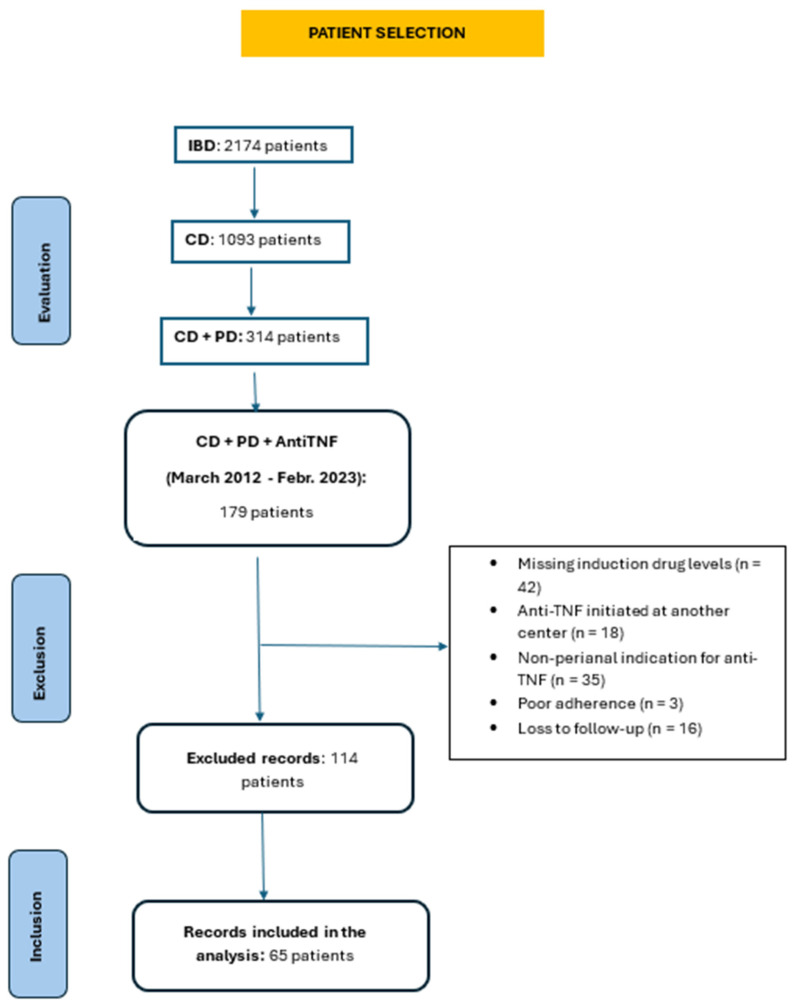
Patient selection flow diagram.

The medical records of 179 patients with CD and PD who had been treated with anti-TNF agents (IFX or ADA) between March 2012 and February 2023 were retrospectively and consecutively reviewed. As shown in the flow diagram (Figure 1), after application of the exclusion criteria, a total of 65 patients were ultimately included in the analysis. All included patients had CD with PD and had initiated anti-TNF therapy exclusively for a perianal indication.

Given the retrospective design, patient inclusion was based on data availability at predefined time points. Patients lost to follow-up or lacking sufficient data were excluded prior to analysis according to the study criteria; therefore, no additional attrition occurred within the analyzed cohort. Treatment regimen modifications during follow-up, including therapy intensification, were specifically recorded and analyzed at week 52.

Patient demographic, IBD-related, and PD characteristics were collected and are detailed in Table 1.

Regarding medical therapy, 47.7% of patients were treated with IFX and 52.3% with ADA. The mean time between the diagnosis of PD and initiation of anti-TNF therapy was 3.55 years (SD 5.51).

A total of 58 patients (89.23%) received treatment with immunomodulators. Among them, 14 patients (24.13%) received immunomodulator monotherapy as first-line treatment, and 26 patients (44.83%) received immunomodulators concomitantly with anti-TNF therapy, with subsequent discontinuation of the immunomodulator. Only 18 patients (31.03%) remained on combination therapy at the end of follow-up.

EUA was performed in 54 patients (82.81%). Of these, 72.22% underwent a two-stage approach, consisting of initial abscess drainage followed by seton placement in a second procedure. Setons were placed in 53 patients (81.5%); 19 patients continued to have setons in situ, while in 34 patients they were subsequently removed.

### 3.2. Clinical and Radiological Outcomes

At week 24, 55 patients (84.61%) achieved a clinical response, of whom 30 (46.15%) were in clinical remission. Among all treated patients, 47.69% of those receiving ADA and 36.92% of those receiving IFX were responders.

Radiological response at week 24 was observed in 30.76% of patients (*n* = 20), of whom 9 were treated with ADA and 11 with IFX. Pelvic magnetic MRI at week 24 was available for 35 of the 65 patients.

During maintenance therapy, at week 52, 80% of patients (*n* = 52) showed a clinical response, with 52.3% achieving clinical remission. Responders comprised 36.92% of patients treated with IFX and 43.08% of those treated with ADA. At 60 months, 10 patients required a switch to a non–anti-TNF therapy. Among patients who continued anti-TNF treatment, 40 patients (61.54%) maintained a clinical response, of whom 33 were in clinical remission. Clinical response at 60 months was observed in 33.85% of patients treated with ADA and 27.69% of those treated with IFX.

Radiological response during maintenance therapy was observed in 52.3% of patients at week 52 (17 treated with ADA and 17 with IFX) and in 46.15% at long term follow-up (60 months; 12 treated with ADA and 16 with IFX). Pelvic MRI during maintenance was available for 40 patients.

No statistically significant differences were observed between IFX and ADA in clinical response at week 24 (χ^2^(1) = 1.56; *p* = 0.21), week 52 (χ^2^(1) = 0.34; *p* = 0.56), or at 60 months (χ^2^(1) = 2.65; *p* = 0.10); nor in radiological response at week 24 (χ^2^(1) = 0.05; *p* = 0.82), week 52 (χ^2^(1) = 0.00; *p* = 1.00), or at 60 months (χ^2^(1) = 0.57; *p* = 0.45).

Regarding treatment intensification at week 52, 35 patients (53.85%) required anti-TNF intensification. Of these, 23 patients (65.72%) were receiving IFX and 12 (34.28%) were receiving ADA.

With respect to treatment persistence at 60 months, 46 patients (70.77%) remained on the same anti-TNF therapy, with 47.83% receiving IFX and 52.17% receiving ADA. Only 5 patients (7.7%) out of the whole cohort developed anti-drug antibodies, all of whom received IFX.

**Figure 2:**
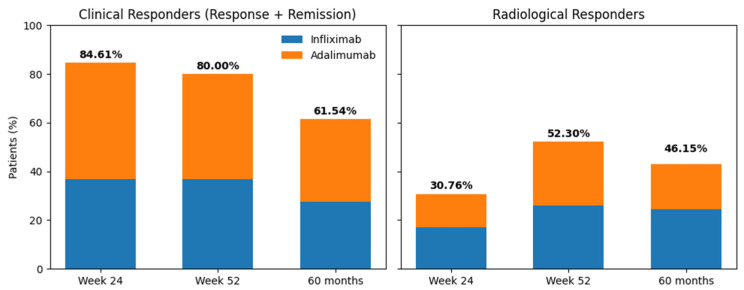
Overall evolution of clinical response and remission (left panel) and radiological response (right panel) at Week 24, Week 52, and 60 months, stratified by anti-TNF therapy.

When evaluating the durability of response, 89.9% of patients who responded at week 52 had already achieved a response at week 24. In addition, 90.7% of early responders maintained response at week 52, with a secondary loss of response rate of 9.3% (Figure 2).

Clinical response at week 24 showed a sensitivity of 89.9% and a specificity of 75% for predicting response at week 52, with a positive predictive value of 90.7% and a negative predictive value of 75%.

When stratified by anti-TNF agent, among patients treated with ADA, 95.8% of week 24 responders maintained response at week 52, compared with 87.5% in the IFX-treated group.

### 3.3. Predictors of Clinical and Radiological Outcomes

To assess the relationship between demographic variables and clinical and radiological response at weeks 24 and 52, a multivariable analysis was performed; the results are shown in Table 2. A statistically significant association was observed between clinical remission at week 24 and the absence of EUA (OR 5.0, 95% CI 0.27–92.7; *p* = 0.004). In addition, a significant association was found between the absence of ANCA positivity and clinical response at week 52 (OR 0.06, 95% CI 0.006–0.59; *p* = 0.01). No statistically significant associations were identified for the remaining variables analyzed.

### 3.4. Anti-TNF Levels and Clinical and Radiological Outcomes

Mean serum anti-TNF levels during induction (weeks 2 and 6) and at week 24 were compared between patients with and without clinical and radiological response at week 52 and at long-term follow-up (60 months) using univariate analyses.

Regarding clinical response at week 52, mean IFX levels at week 2 were 25.8 µg/mL (SD 4.1) in non-responders and 30.9 µg/mL (SD 14) in responders (*p* = 0.39). At week 6, mean levels were 17.2 µg/mL (SD 12.2) and 19.4 µg/mL (SD 13.8), respectively (*p* = 0.70). Mean ADA levels at week 2 were 13.3 µg/mL (SD 7.7) in non-responders and 14.0 µg/mL (SD 6.3) in responders (*p* = 0.87). At week 6, ADA levels were 10.1 µg/mL (SD 3.3) in non-responders and 12.0 µg/mL (SD 6.1) in responders (*p* = 0.59).

With respect to radiological response at week 52, mean IFX levels at week 2 were 27.0 µg/mL (SD 15.3) in non-responders and 32.7 µg/mL (SD 14.5) in responders (*p* = 0.45). At week 6, mean levels were 15.9 µg/mL (SD 6.7) and 23.7 µg/mL (SD 14.8), respectively (*p* = 0.27). In the ADA-treated group, mean week-2 levels were 14.8 µg/mL (SD 7.6) among responders; only one patient failed to achieve radiological response at this time point. At week 6, ADA levels were 12.3 µg/mL (SD 5.9) in non-responders and 12.7 µg/mL (SD 6.2) in responders (*p* = 0.94).

**Figure 3:**
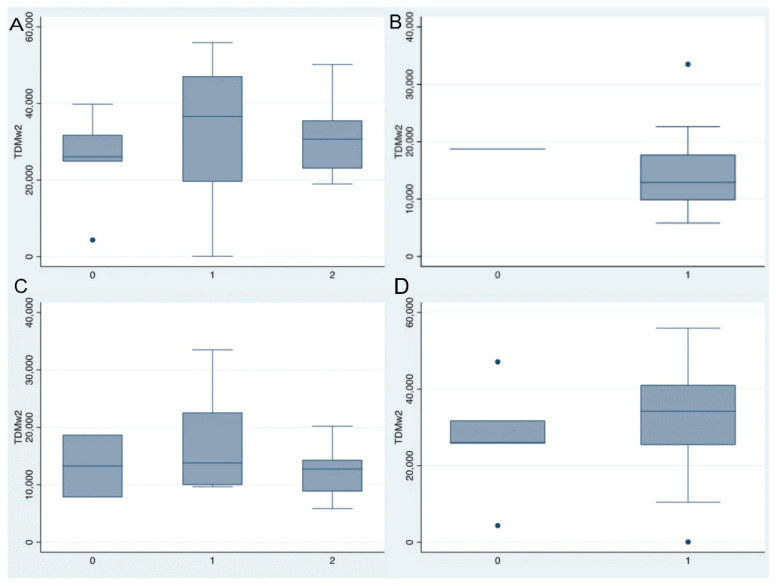
(**A**) Infliximab levels at week 2 and clinical response at week 52 (0 = no response, 1 = response, 2 = remission). (**B**) Infliximab levels at week 2 and radiological response at week 52 (0 = no response, 1 = response). (**C**) Adalimumab levels at week 2 and clinical response at week 52 (0 = no response, 1 = response, 2 = remission). (**D**) Adalimumab levels at week 2 and radiological response at week 52 (0 = no response, 1 = response).

The distribution of IFX and ADA levels at week 2 according to clinical and radiological response at week 52 is shown in Figure 3.

In the long-term maintenance analysis (60 months), mean IFX and ADA levels during the induction period (weeks 2 and 6) and at week 24 were collected, as shown in Table 3. In the IFX-treated group, a trend toward higher clinical and radiological response rates was observed at weeks 6 and 24 with increasing drug concentrations, although statistical significance was not reached. However, this trend was not maintained during the maintenance phase. No correlation was observed at any time point in patients treated with ADA.

An estimation of optimal anti-TNF levels during induction (weeks 2 and 6) was performed to assess their ability to predict both radiological and clinical response at week 52. ROC curve analyses indicated no meaningful discriminative capacity of drug levels during induction for long-term outcomes, with AUC values consistently close to 0.5.

For radiological response, IFX levels of 8.79 µg/mL at week 2 yielded a sensitivity of 94.12%, with low AUC (0.447). At week 6, IFX levels of 8.2 µg/mL were associated with radiological response, with a sensitivity of 88.24% and an AUC of 0.529.

Regarding clinical response, at week 2, an IFX concentration of 19.01 µg/mL was associated with clinical response, with a sensitivity of 81.82% and an AUC of 0.537. At week 6, an IFX level (4.73 µg/mL) was associated with clinical response, showing a sensitivity of 86.96% and an AUC of 0.456.

For ADA, radiological response at week 52 was associated with drug levels of 9.97 µg/mL at week 2 (sensitivity 87.5%, AUC 0.513) and 8.87 µg/mL at week 6 (sensitivity 73.33%, AUC 0.533).

In terms of clinical response, an ADA level of 8.41 µg/mL at week 2 was associated with response at week 52, with a sensitivity of 86.96% but a low AUC (0.376). At week 6, ADA levels of 8.87 µg/mL were associated with clinical response, with an AUC of 0.583 and a sensitivity of 74.07%.

## 4. Discussion

The present findings should be interpreted within the framework of a long-standing assumption that perianal disease necessitates higher serum anti-TNF levels compared with luminal disease. This concept, widely adopted in clinical practice, is largely derived from retrospective studies and heterogeneous cohorts in which the indication for treatment was not always strictly perianal, clinical and radiological assessments did not follow uniform criteria, and only drug levels were considered, while other key components of multidisciplinary management were overlooked. Consequently, the recommendation to pursue higher drug levels in PD has become established more as a pragmatic interpretation than as a conclusion supported by robust evidence.

The pioneering studies that demonstrated the efficacy of IFX in perianal fistulas, such as the trial by Present et al. [9] and its subsequent evaluation in the ACCENT II study [10], provided essential information supporting the use of this agent in PD. Similarly, the CHARM trial confirmed the utility of ADA for fistula closure and maintenance of fistula response [11]. However, subsequent studies proposing the need for higher serum drug levels in this clinical phenotype have frequently been based on small sample sizes, broad inclusion criteria, or mixed populations with both luminal and perianal indications [12,15,16,17]. In addition, many of these studies did not incorporate systematic RMI or homogeneous definitions of remission, thereby limiting the extrapolation of their findings. Despite these methodological limitations, such recommendations have been widely incorporated into current clinical guidelines [18].

In contrast, the present study applies more stringent inclusion criteria by considering only patients in whom anti-TNF therapy was initiated specifically for PD, uses structured radiological assessment as part of follow-up, and evaluates long-term outcomes while taking into account drug levels during both induction and maintenance phases. Under these conditions, the analyses did not demonstrate statistically significant associations between higher serum drug levels and an increased likelihood of clinical or radiological response at any of the phases analyzed, either during induction or maintenance.

In our study, a statistically significant association was observed between clinical remission at week 24 and the absence of EUA, suggesting a selection bias, with patients not undergoing the procedure likely representing those with milder disease.

Pivotal trials [11] and cohorts such as those described by Panés and colleagues [17,19] have shown a more linear relationship between drug concentration and response. Nevertheless, these studies were conducted in highly controlled clinical trial settings, with less anatomical heterogeneity, lower fistula burden, and without the complexity of real-world factors such as surgical interventions during treatment.

These findings call into question the assumption that universally higher drug levels are required as a therapeutic target in PD and emphasize the need for more methodologically robust studies.

In addition, our study showed that an early disease course was directly associated with long-term response. Specifically, 90.7% of early responders maintained their response at week 52, with a secondary loss of response rate of 9.3%, suggesting that the absence of early improvement may represent a clinically useful marker for anticipating a less favorable outcome. This observation, emerging from a homogeneous cohort, raises the possibility that early treatment failure could justify consideration of alternative therapeutic strategies. In this context, the growing availability of agents with different mechanisms of action, such as JAK inhibitors [20,21] or IL-23 antagonists [22], offers potential options for patients who fail to show improvement during the early phases of anti-TNF therapy. Although this approach requires prospective validation, our data suggest that early response assessment may play a relevant role in optimizing therapeutic decision-making in PD.

The main limitations of our study include its retrospective design, the relatively small sample size, the unequal availability of MRI at all time points, and its single-center nature. Given the retrospective nature of the study, imaging assessments were not standardized nor performed at predefined time points for all patients. MRI examinations were requested according to routine clinical practice, which may have introduced selection bias in the radiological subgroup analyses.

Furthermore, although response definitions are not standardized, we applied those most frequently used in the literature following a thorough review of the evidence in this field. Another limitation relates to the number of outcome events relative to the covariates included in the multivariate model. Although variables were selected based on clinical relevance and prior evidence, the limited events-per-variable ratio may increase the risk of model overfitting. Therefore, the results of the adjusted analysis should be interpreted with caution and confirmed in larger prospective studies.

Regarding the chronology of seton management, including placement and removal, institutional protocol dictates that setons are not removed before week 24; therefore, the potential impact of early seton removal was not assessed. Likewise, the influence of EUA prior to initiation of biological therapy was not evaluated, as most included patients had undergone more than one EUA.

Despite these limitations, the study provides clinically relevant data from a cohort in which anti-TNF therapy was initiated exclusively for PD, contributing to a more refined interpretation of the available evidence and allowing better contextualization of the role of pharmacokinetic monitoring, including in the long term.

Given that the present study represents one of the largest real-world cohorts of patients with PD treated with anti-TNF agents, several relevant conclusions can be drawn. First, we confirm that PD remains a marker of aggressive disease and therapeutic difficulty, in line with previous studies [7,8,9,10,11,12,13,14]. The high proportion of complex fistulas reflects the characteristics of patients managed in specialized units and partly explains the frequent need for treatment intensification, as described by Bouguen et al. [13] and Makowiec et al. [23].

Regarding the role of anti-TNF levels, we observed that higher trough levels during induction were not statistically associated with improved clinical or radiological response in a cohort in which the indication for treatment was exclusively PD. This finding challenges the simplified interpretation that higher drug levels invariably lead to sustained fistula closure. In contrast, early response was clearly associated with long-term outcomes. Although these results are encouraging, they should be interpreted with caution, given the retrospective design and the limited sample size.

Our cohort highlights the limitations of isolated therapeutic drug monitoring as a decision-making tool in PD. Prospective studies integrating biomarkers, microbiota, genetic factors, and anatomical characteristics are needed to develop robust predictive models.

## Figures and Tables

**Table 1:** Demographic, Crohn’s disease–related, and perianal disease characteristics of the study population.

**Characteristic**	**Value**
Age (years), mean (SD)	46.28 (14.62)
Sex, male/female (% male)	30/35 (46,2%)
Body mass index (kg/m^2^) mean (SD)	25.57 (5.09)
Current smoker, N (%)	21 (32.3%)
Former smoker, N (%)	15 (23.1%)
Never smoker, N (%)	29 (44.6%)
Extraintestinal manifestations, N (%)	24 (36.9%)
Disease Location (L1/L2/L3), N (%)	10 (15.4%)/32 (49.2%)/23 (35.4%)
Disease behavior (B1/B2/B3), N (%)	35 (53.8%)/12 (18.5%)/18 (27.7%)
Previous surgery before Anti-TNF	
No, N (%)	21 (32.3%)
Yes, N (%) ▪ Ileocecal resection ▪ Right hemicolectomy ▪ Perianal surgery	44 (67.7%) 9 2 39
Treatment prior to Anti-TNF therapy	
▪ Thiopurines	61 (93.8%)
▪ Corticosteroids	29 (44.6%)
▪ Biologic agents	23 (35.4%)
Anti-TNF therapy	
▪ Infliximab (IFX)	10 (15.4%)
▪ IFX biosimilar	22 (33.8%)
▪ Adalimumab (ADA)	26 (40%)
▪ ADA biosimilar	7 (10.8%)
Time from CD diagnosis to onset of PD	
<1 year	28 (43.1%)
1–5 years	14 (21.5%)
>5 years	23 (35.4%)
Fistula type	
Simple, N (%)	12 (18.5)
Complex, N (%)	53 (81.5)
Parks classification	
Superficial, N (%)	6 (9.38)
Intersphincteric, N (%)	9 (14.06)
Transsphincteric, N (%)	43 (66.15)
Suprasphincteric, N (%)	5 (7.81)
Extrasphincteric, N (%)	2 (3.12)
Number of fistulous tracts	
One, N (%)	32 (48.65)
Two, N (%)	12 (18.75)
Three, N (%)	12 (18.75)
Four or more, N (%)	9 (13.85)
Rectovaginal fistulas	
Yes, N (%)	9 (13.85)
No, N (%)	56 (86.15)
Associated abscess	
Yes, N (%)	58 (89.23)
No, N (%)	7 (10.77)

**Table 2:** Multivariate analysis of predictive factors of clinical and radiological response at weeks 24 and 52.

Factor	Clinical Response									Radiological Response					
	Week 24			Week 52			60 Months			Week 24			Week 52		
	OR	Mean (CI95%)	*p* Value	OR	Mean (CI95%)	*p* Value	OR	Mean (CI95%)	*p* Value	OR	Mean (CI95%)	*p* Value	OR	Mean (CI95%)	*p* Value
**Sex**	1.52 (0.18–12.89)	-	0.69	0.89 (0.13–5.97)	-	0.91	0.69 (0.16–2.99)	-	0.62	4.67 (0.78–27.9)		0.09	0.82 (0.07–9.33)		0.90
**Age**	-	38.6 ± 12.8 vs. 47.7 ± 14.6	(−19.4–1.3)	-	45.8 ± 14.2 vs. 46.7 ± 14.5 (−10.5–8.7)	0.85	–	-	–		47.8 ± 12.9 vs. 52.3 ± 13.7 (−6.7–15.7)	0.41		50.4 ± 11.9 vs. 49.1 ± 16.4 (−12.5–12.9)	0.83
**Weight**	-	63.9 ± 12.2 vs. 73.6 ± 16.7 (−21.3–2.1)	0.11		77.1 ± 22.2 vs. 71.7 ± 14.8	(−5.4–16.2)	–	-	–		79.7 ± 16.6 vs. 68.1 ± 19.3 (−26.8–3.7)	0.11		72.5 ± 16.1 vs. 76.4 ± 19 (−22.6–14.4)	0.60
**Ileal/ileocolic location**	0.79 (0.07–8.32)	-	0.84	1.21 (0.30–4.92)		0.78	0.97 (0.34–2.76)	-	0.96	–	–	–	–	–	–
**ANCA**	0.58 (0.04–7.129	-	0.74	0.06 (0.006–0.5)	-	0.01	0.86 (0.05–12.62)	-	0.91	–	–	–	–	–	–
**Extraintestinal manifestations**	3.45 (0.32–37.28)	-	0.30	0.68 (0.09–5.09)	-	0.71	0.38 (0.08–1.77)	-	0.22	0.77 (0.18–3.29)		0.74	0.94 (0.33–2.69)	–	0.89
**Current smoker**	1.16 (0.26–5.21)	-	0.25	1.16 (0.35–3.85)	-	0.80	0.83 (0.33–2.12)	-	0.71	0.48 (0.07–3.21)		0.39	0.79 (0.09–6.67)	–	0.97
**Time from diagnosis to anti-TNF therapy**	-	2.33 ± 3.27 vs. 3.65 ± 5.8 (−5.4–2.8)	0.51	-	3.36 ± 4.36 vs. 3.71 ± 5.83 (−4.7–4.0)	0.85	–	–	–	–	3.88 ± 6.11 vs. 1.35 ± 1.72 (−0.5–5.6)	0.09	–	1.37 ± 1.5 vs. 3.58 ± 5.73 (−7.1–2.7)	0.28

**Table 3:** Relationship between long-term clinical and radiological response and mean IFX and ADA levels at weeks 2, 6, 24, and 52.

	Week 2: Mean Serum IFX Concentration, µg/mL (SD)	*p* Value	Week 6: Mean Serum IFX Concentration, µg/mL (SD)	*p* Value	Week 24: Mean Serum IFX Concentration, µg/mL (SD)	*p* Value	Week 52: Mean Serum IFX Concentration, µg/mL (SD)	*p* Value
**Clinical response at 60 months**		0.8		0.58		0.36		0.78
			
**No**	30.16 (16.02)		17.52 (10.12)		5.1 (5.5)		9.7 (8.35)	
**Yes**	28.59 (13.88)		20.62(14.85)		7.33 (6.03)		8.87 (6.67)	
**Radiological response at 60 months**		0.56		0.27		0.17		0.84
			
**No**	24.13 (17.78)		12.81 (8.4)		2.58 (1.92)		9.34 (10.23)	
**Yes**	28.97 (14.29)		21.76 (15.17)		7.06 (5.89)		10.19 (6.97)	
	**Week 2: Mean serum ADA concentration,** **µg/mL** **(SD)**	***p*** **Value**	**Week 6: Mean serum ADA concentration,** **µg/mL** **(SD)**	***p*** **Value**	**Week 24: Mean serum ADA concentration,** **µg/mL** **(SD)**	***p*** **Value**	**Week 52: Mean serum ADA concentration,** **µg/mL** **(SD)**	***p*** **Value**
**Clinical response at 60 months**		0.97		0.45		0.56		0.83
			
**No**	14.67 (7.45)		13.36 (5.33)		9.19 (8.04)		10.27 (9.55)	
**Yes**	14.53 (6.95)		11.21 (6.28)		10.84 (5.6)		10.85 (4.71)	
**Radiological response at 60 months**		0.74		0.35		0.08		0.65
			
**No**	15.94 (6.89)		10.26 (3.49)		6.16 (4.9)		9.92 (9.4)	
**Yes**	14.39 (8.62)		13.42 (7.6)		11.27 (5.55)		11.55 (5.69)	

## Data Availability

The data presented in this study are not publicly available due to ethical and privacy restrictions related to patient confidentiality. Data are available from the corresponding author upon reasonable request and with permission of the institutional ethics committee.

## Abbreviations

The following abbreviations are used in this manuscript:

ADA	Adalimumab
ANCA	Antineutrophil Cytoplasmic Antibodies
ASCA	Anti-Saccharomyces cerevisiae Antibodies
AUC	Area Under the Curve
CD	Crohn’s Disease
CI	Confidence Interval
EFO	External Fistula Opening
ELISA	Enzyme-Linked Immunosorbent Assay
EUA	Examination Under Anesthesia
IBD	Inflammatory Bowel Disease
IFX	Infliximab
MRI	Magnetic Resonance Imaging
OR	Odds Ratio
PD	Perianal Disease
ROC	Receiver Operating Characteristic
SD	Standard Deviation
TNF	Tumor Necrosis Factor
